# SPINGO: a rapid species-classifier for microbial amplicon sequences

**DOI:** 10.1186/s12859-015-0747-1

**Published:** 2015-10-08

**Authors:** Guy Allard, Feargal J. Ryan, Ian B. Jeffery, Marcus J. Claesson

**Affiliations:** School of Microbiology and APC Microbiome Institute, University College Cork, Cork, Ireland

**Keywords:** Microbiota composition, Metagenomics, Species-classification, 16S rRNA gene amplicons, *cpn60*

## Abstract

**Background:**

Taxonomic classification is a corner stone for the characterisation and comparison of microbial communities. Currently, most existing methods are either slow, restricted to specific communities, highly sensitive to taxonomic inconsistencies, or limited to genus level classification. As crucial microbiota information is hinging on high-level resolution it is imperative to increase taxonomic resolution to species level wherever possible.

**Results:**

In response to this need we developed SPINGO, a flexible and stand-alone software dedicated to high-resolution assignment of sequences to species level using partial 16S rRNA gene sequences from any environment. SPINGO compares favourably to other methods in terms of classification accuracy, and is as fast or faster than those that have higher error rates. As a demonstration of its flexibility for other types of target genes we successfully applied SPINGO also on *cpn60* amplicon sequences.

**Conclusions:**

SPINGO is an accurate, flexible and fast method for low-level taxonomic assignment. This combination is becoming increasingly important for rapid and accurate processing of amplicon data generated by newer next generation sequencing technologies.

**Electronic supplementary material:**

The online version of this article (doi:10.1186/s12859-015-0747-1) contains supplementary material, which is available to authorized users.

## Background

Analysis of microbial communities (microbiota) sampled directly from their natural environment, without clonal culturing, is a rapidly evolving field with wide-ranging applications within ecology, agriculture and medicine. A relatively straight-forward approach for characterizing and comparing microbiota is to sequence variable regions of the ubiquitous 16S rRNA gene following amplification using universal primer pairs. The resulting sequence reads can either be analysed as groups of similar sequences (operational taxonomic units: OTUs), or as raw reads. In either case, taxonomic classification of the resulting sequence reads is a crucial component for characterising microbiota composition. The most common tool for this is the RDP-Classifier which generally assigns partial 16S rRNA gene sequences down to genus level [1]. There is, however, a need among investigators to increase the taxonomic resolution to include species assignments wherever possible. For example, a genus like *Streptococcus* has species that are either considered beneficial (*S. thermophilus*) or pathogenic (*S. pneumoniae*), thus it is crucial to be able to identify species with good accuracy whenever sequence specificity allows it. In addition, a subset of the Gram-positive and endospore-forming bacterial species have traditionally been structured into *Clostridium* clusters, primarily based on 16S rRNA gene similarities [2]. Many of the species in these clusters often belong to genera other than *Clostridium*, often due to discrepancies between their traditionally characterised phenotypes and molecular phylogeny. As there are established primer combinations for many of these clusters, which are frequently used by microbiologists to elucidate microbiota community structure, there is a need to link high-throughput data derived from culture-independent methods to these more targeted and traditional methods.

So far, the few existing methods that can be used for species classification “out-of-the-box” are rather limited and not designed for such purposes: they are either applied on a very restricted set of species [3, 4], or are only suitable on reads from soon-obsolete technologies like the 454 Pyrosequencing due to the low computational classification speed [5]. Even though broad taxonomic assignment of representative OTU sequences is the main objective for UCLUST as implemented by the *assign_taxonomy.py* script within the QIIME software suite [6], it does have the capacity for species-level assignment when Greengenes is used as a reference database [7]. However, this is just for a minor subset of OTUs as Greengenes only have 627 unique species (version 13.5) compared to 12,394 species in the RDP database (version 11.2) compliant with the NCBI Taxonomy. While both databases have uneven representation of taxa, this is more prominent for Greengenes where the most abundant species is *Faecalibacterium prausnitzii* (15 % of sequences with species classification) compare to the RDP database where the most abundant species is *Bacillus subtilis* (2 %). Both the Java and mothur implementations of the RDP-classifier can also be used for species classifications, however these methods were designed for broader taxonomic classification [1] and require re-training using non-default databases. A versatile species-classifier should be able to classify sequenced from very diverse environments, and also be capable of efficiently processing millions of amplicon sequences generated by more contemporary and low-cost high-throughput technologies, e.g. Illumina MiSeq, within a reasonable time-frame. This sequencing technology now routinely generates 300 bp long paired-end reads, thereby facilitating coverage of several adjacent variable regions of the 16S rRNA gene when overlapping paired-end reads are merged.

Here, we present SPINGO (Species-level IdentificatioN of metaGenOmic amplicons), a stand-alone software application capable of classifying assignable species sampled from any environment. Its flexible design, accuracy and speed allows for frequent taxonomy updates facilitating even more precise high-resolution classifications without becoming a computational bottleneck for downstream analysis.

## Implementation

### Construction of a species reference database

Full-length (≥1200 bp) bacterial and archaeal 16S rRNA gene sequences were obtained from the Ribosomal Database Project version 11.2 (http://rdp.cme.msu.edu/). All sequences were labelled to species names according to the NCBI (http://www.ncbi.nlm.nih.gov/guide/taxonomy/), which is readily available and distributes the original nomenclature as deposited with the submitted sequence (http://www.ncbi.nlm.nih.gov/WebSub/html/requirements.html). Only sequences with complete binomial (genus + species) names were retained, and identical sequences from the same species were removed in order to reduce the training dataset. Sequences that were identical, but associated to multiple species were on the other hand retained, as such sequences represent species that are not assignable using our algorithm outlined below. Thus, the resulting SPINGO reference database only contained full-length, species-specific 16S rRNA gene sequences, which were non-redundant for each species. For example, if Species A has sequences ACG/ACC/ACC/CCC before this operation, it will afterwards only have sequences ACG/ACC/CCC. From this SPINGO database of 95,210 sequences and 12,394 unique species, a taxonomy mapping file was created linking the original sequence identifiers with a two-level hierarchy comprising both genus and species names, as well as *Clostridium* clusters where applicable. For the latter, a lookup table linking species names with these clusters had previously been compiled [2, 8]. Albeit not the main aim of SPINGO, genus-level classification is also enabled by default to broaden its application for high taxonomic resolution. The taxonomy mapping file can be re-used by the *make_database.py* script to facilitate future updates or reconstruction based on other types of taxonomic hierarchies.

### Algorithm

Assignment of amplicons to the closest known species is based on the reference database described above. This database is loaded into memory and indexed by *k-mers* using an inverted index structure, a

$$ {S}_{Q,R}=\frac{\left|{Q}_K\cap {R}_K\right|}{\left|{Q}_K\right|} $$

commonly used index data structure for storing words (*k-mers*), which allows for rapid retrieval of all sequences which contain a given word (*k-mer*). Query sequences are then compared to the reference database using the following similarity score: given a query sequence Q and a reference sequence R, Q_K_ is the set of overlapping and fixed length *k-mers* present in Q, and R_K_ is the set of overlapping *k-mers* in R. A similarity score S_Q,R_ is calculated as the number of *k-mers* shared between the reference and query sequence, normalized by the number of unique *k-mers* in the query sequence thus giving a number in the range [0, 1].

For each query sequence, the database is searched using both the forward and reverse complement of the query and a list of the reference sequence(s) giving the highest score is retrieved. For each of the taxonomic levels in the two-level hierarchy, genus and species level, as well as clostridium cluster, an assignment is made at that level if the annotations of the reference sequences are in agreement, otherwise the assignment is considered to be ambiguous. If an assignment is made at any taxonomic level, a bootstrapping process, similar to that of the RDP-classifier [1], is performed to provide a confidence estimate of the taxonomic assignment. Briefly, for each bootstrap trial at a given *k-mer* size k_size_, a subset q_k_ of Q_K_ is sampled at random, where |q_k_| = |Q_k_|/k_size_. The taxonomic annotations at each level for the reference sequences giving the highest S_q,R_ are obtained. The confidence estimate is then calculated as the proportion of retrieved sequences with a taxonomic assignment matching that of Q_k_ at the same level. A low confidence estimate indicates that many reference sequences have a similar (but not identical) set of *k-mers* (low distinctiveness), while a high confidence estimate indicates that there are few reference sequences with a similar composition (high distinctiveness).

### Creation of validation datasets

To evaluate SPINGO and demonstrate its utility for species classification we used two different approaches. First, we used a 10-fold cross validation [9] with the SPINGO database on four different methods for species classification: SPINGO, the mothur-implementation of the RDP-classifier (v1.34.1), UCLUST (v1.2.22q; default method in QIIME’s *assign_taxonomy.py*) and BLASTn (v.2.2.28), while keeping database, *k-mer* size (8-mer) and number of bootstrap runs (100) constant across compared methods. All these methods use enumeration of *k-mers* at an early stage, but differ significantly in how these counts are processed in the downstream analysis. A key difference between SPINGO and the other algorithms is that SPINGO identifies sequences for indistinguishable species and discards them as ambiguous candidates, whereas the other methods will always classify the query sequence even if there are multiple conflicting hits. Even so, by specifying a non-default option it is also possible to list all ambiguous species hits. SPINGO is thus designed to classify relatively short sequences where the percentage deviation from a reference sequence is relatively small. One can view *k-mer* counting as a proxy for standard pairwise sequence alignment based on sequence similarity, but as there still are some important differences it can be useful to briefly outline situations where false positive and negatives will occur. For example, if a sequence is made of two regions A = ATATTAAATT and B = GCCGGGCGGC the *k-mers* would be ATAT TATT ATTA TTAA TAAA AAAT AATT ATTG TTGC TGCC GCCG CCGG CGGG GGGC GGCG GCGG CGGC, while if A and B where switched the *k-mers* would be GCCG CCGG CGGG GGGC GGCG GCGG CGGC GGCA GCAT CATA ATAT TATT ATTA TTAA TAAA AAAT AATT, with the *k-mers* unique to either A or B underscored. Thus, the *k-mer* similarity score would be high (14/17), but an alignment score would be low resulting in a false positive. A similar situation could occur at the start or end of a sequence: For example, if there is a substitution at the start of sequence 5′-ATTTGCG, which has *k-mers* ATTT TTTG TTGC TGCG, to 5′-GTTTGCG the new *k-mers* are GTTT TTTG TTGC TGCG, resulting in a *k-mer* similarity score of 3/4 against the original sequence. However, if there instead is a substitution in the middle to 5′-ATTCGCG the new *k-mers* are ATTC TTCG TCGC CGCG, resulting in *k-mer* similarity score of 0, much lower than an alignment score (false negative). False negatives will also occur if a query sequence contains a large number of errors equally spread along the sequence, as the *k-mer* score will be lower than what a global alignment score would be. Nevertheless, a sequence that is not classified due to a large number of mutations or sequencing errors should not be classified, even if there is a high global similarity. This makes sense in a situation where different species may differ in only a small number of bases. So while these issues are worth considering, our empirical data shows that they do not adversely affect the classifier performance. False positive rate will be more greatly affected by mislabelled sequences in the database. As for false-negatives, SPINGO does not try to predict which species are not in a sample - absence of evidence is not evidence of absence - so that discussion is purely academic.

For each 10^th^ of the SPINGO database, 12 different variable 16S rRNA gene regions were extracted using the V-ripper script (Additional file 1 and GitHub distribution) and classified. Second, we obtained three different datasets, based on a simulation, a mock community and a real-life environmental sample. For the simulation, we created a dataset of 10,067 full-length 16S rRNA gene sequences, each representing one type strain, from the SILVA Living Tree Project version 11.5 [10] using the NCBI Taxonomy nomenclature,. This facilitated a like-for-like comparison with the SPINGO database which contains sequences from the RDP database, but with species names labelled according to the NCBI Taxonomy. A hold-out evaluation database was created by removing 9,607 sequences from the SPINGO database that were present in the SILVA database. Variable regions V1-V3 (6,046 sequences), V3-V5 (5,860) and V6-V9 (5,241) were extracted from the SILVA database using previously described primers [11] with the V-ripper script and subsequently classified using the evaluation database not containing the 9,607 test sequences. In addition, we classified sequences derived from a mock community of 21 known bacterial species in even composition [12]. The 454 Pyrosequencing reads covering the hyper-variable regions V1-V3, V3-V5 and V6-V9 were chimera filtered using UCHIME [13] with the “Gold” database (http://microbiomeutil.sourceforge.net) as reference to remove chimeric sequences. Sequences were considered to be correctly classified if the unambiguously assigned species was a known component of the mock community. To also explore a real biological environment we analyzed amplicon sequences based on the three primer combinations referred to above for a stool sample originating from a healthy male subject (sample SRS019089 from the Human Microbiome Project http://hmpdacc.org/HM16STR/healthy).

SPINGO’s accuracy and target versatility was finally demonstrated and evaluated on amplicon sequences derived from the universal house-keeping gene cpn60. Here, a 10-fold cross validation was performed on 6,690 amplicon sequences of the cpn60 Universal Target region (~500 bp) for which there was a full species name, which were downloaded from cpnDB [14] on March 4^th^ 2015 (http://www.cpndb.ca/search.php). The scripts and syntax used for evaluation are available in the Additional file 1.

## Results and discussion

### Performance evaluation

**Fig. 1:**
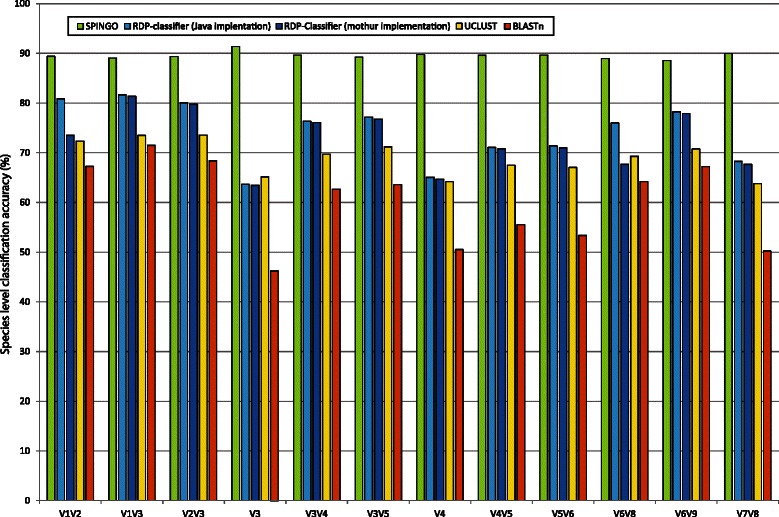
Comparison of species level classification accuracy for 12 different 16S rRNA gene regions by SPINGO, RDP-Classifier, UCLUST and BLASTn, using 10 fold cross validation. All classifiers were trained on the SPINGO 16S species level database, used *k-mer* size 8 and 100 bootstraps

The 10-fold cross validation found SPINGO to consistently have the highest classification accuracy regardless of 16S rRNA gene region (Fig. 1), when keeping *k-mer* sizes, database and number of bootstraps constant. Across all 16S rRNA gene regions tested, SPINGO provided an increase of on average 15 % to 17 % percentage points in classification accuracy over the two RDP-classifiers, 21 percentage points over UCLUST, and 30 % percentage points over BLASTn. Interestingly, while both implementations of the RDP-classifier give comparable accuracies for most regions, the Java implementation performs better than the mothur implementation for the V1V2 and V6V8 regions. The 10-fold cross validation also demonstrates that SPINGO’s classification accuracy is less impacted by sequence length than the other classifiers, as can be observed for the shorter regions. We also investigated whether the accuracy of SPINGO varied much for the various combinations of *k-*mer sizes and number of bootstraps. There were only marginal differences where the average accuracy for 8-mers was 0.5 % higher with 10 times more bootstrap runs, whereas it was 1.9 % higher for 12-mers for the corresponding bootstrap increase (Additional file 2: Figure S1).

**Fig. 2:**
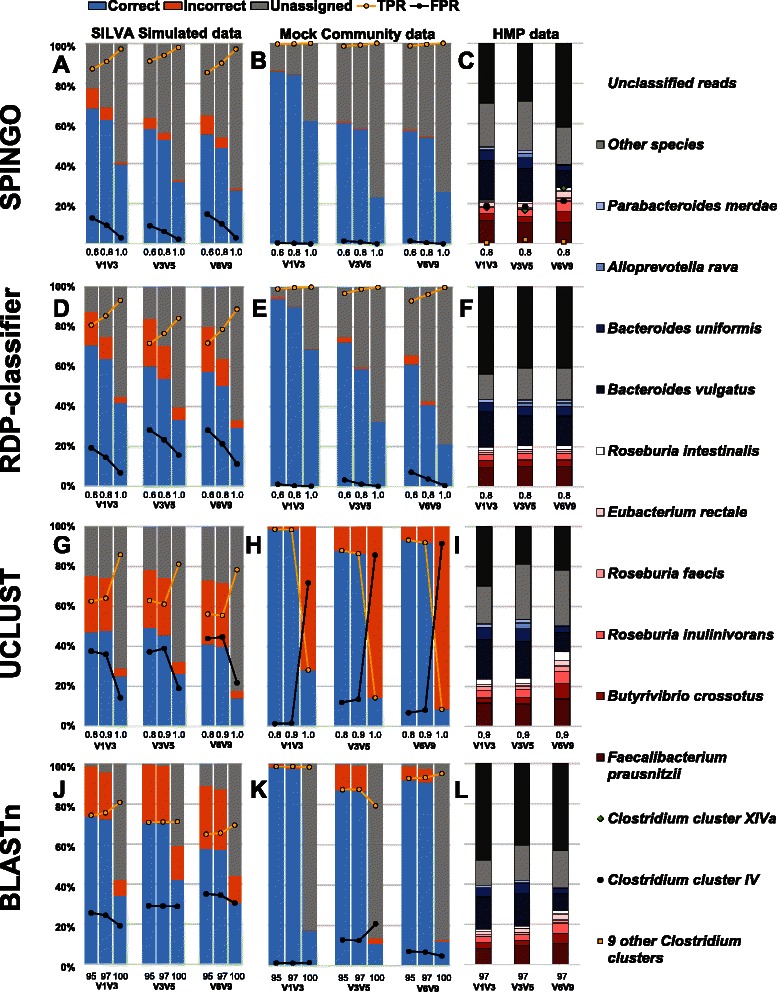
Performance of SPINGO across three different datasets and three amplicon regions (8-mers with 100 sub-samples; confidence estimate cut-offs at the X axis). **a** Species classification of the SILVA sequences, and **b** 21 bacterial species from a mock community. Proportion of correctly TPR = TP/(TP + FP), and incorrectly FPR = FP/(TP + FP) assigned sequences. **c** Stacked relative species abundance and un-stacked proportions of the most abundant Clostridium clusters in a single stool sample. Species from the Clostridiales order as red gradient and Bacteroidales order as blue gradient. Corresponding comparisons for the mother implementation of the RDP-Classifier (**d**-**f**), UCLUST (**g**-**i**; X axis shows UCLUST similarity cut-offs), and BLASTn (**j**-**l**; X axis shows Percent identity). All classifiers were trained on the SPINGO database, using *k-mer* size 8 and 100 bootstraps

The second evaluation approach involved the simulated, mock and real-life samples, using the four different methods, all applied on SPINGO’s database: SPINGO (Fig. 2a-c); the RDP-classifier (Fig. 2d-f); UCLUST (Fig. 2g-i); and BLASTn (Fig. 2j-l). Unsurprisingly, the proportion of incorrect assignments decreased with higher confidence estimate cut-offs. The higher proportion of incorrect assignments in the SILVA dataset can be attributed to its greater species diversity. For this dataset, SPINGO provided the highest True Positive Rate of any of the classifiers across the three regions tested, and while other classifiers such as BLASTn may classify a higher proportion of sequences SPINGO makes far fewer mistakes, in agreement with the results from the 10-fold cross validation. Similarly for the 21-strain mock community, SPINGO provides the best True Positive Rate across the regions and confidence estimates, albeit the difference between SPINGO and the RDP-classifier here is smaller. Both BLASTn and UCLUST classified more sequences than SPINGO and the RDP-classifier, but at a severe cost to accuracy, except for the V1V3 region where UCLUST and BLASTn were comparable to SPINGO for the lower similarity cut-offs and percent identities, respectively. For the real-life HMP sample there was relatively little variation of microbiota composition for the 10 most prevalent species, with SPINGO classifying marginally fewer species than the other methods. Depending on the variable region, roughly half of the reads were assigned to a species, whereof the 10 most abundant species have been associated to the human gastrointestinal tract (Fig. 2c). Given SPINGO’s higher classification accuracy from the 10-fold cross validation and consistently higher True Positive Rate over the simulated and mock datasets tested, much due to not accepting ambiguous hits, it is quite conceivable that the SPINGO assignments are more often correct. Interestingly, the V1V3 region consistently shows the greatest accuracy of all three 16S regions. The by SPINGO assigned *Clostridium* clusters are an additional and useful feature for researchers interested in the gut microbiome. Note that while a sequence can be unassigned at species level due to ambiguity it may still be unambiguously assigned to a *Clostridium* cluster, which explains the larger proportions at this level.

### Species classification of non-16S rRNA gene sequences

**Fig. 3:**
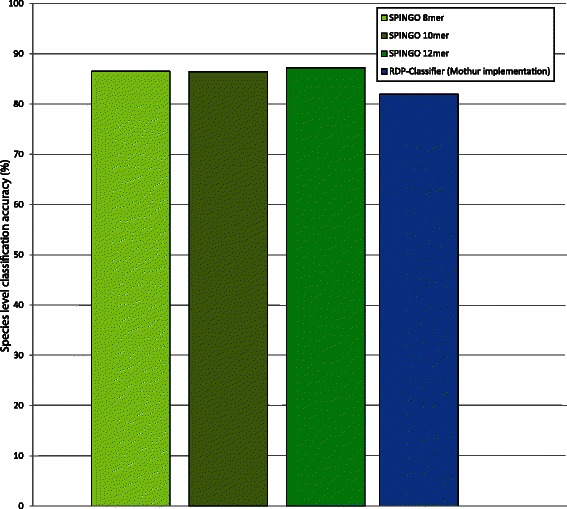
Species level classification accuracy for SPINGO (*k-mer* sizes 8,10 and 12, and 100 sub-samples) and RDP-Classifier (only *k-mer* size 8 and 100 sub-samples due to RAM exhaustion for higher k-mer sizes) as assessed by 10-fold cross validation on a database of 6,690 cpn60 sequences, using the Universal Target region of each sequence for classification

To finally illustrate how SPINGO can be applied for species-classification using other types of sequences than 16S rRNA genes, we trained both SPINGO and the mothur-implementation of the RDP-classifier on cpnDB data (see Implementation), which the latter method has been used for previously [14]. This single-copy gene has been used as an alternative target for amplicon sequences due to its high resolution at species level. The accuracy of SPINGO and the RDP-classifier for assigning sequences from the cpn60 Universal Target region, an alternative to the 16S rRNA gene hyper-variable regions, was also here assessed by performing a 10-fold cross validation. As with the results from the 16S validation, SPINGO once again shows higher classification accuracy than the RDP-classifier (Fig. 3) for three different *k-mer* sizes, although the increase is less pronounced with 4.6 percentage points.

### Computational performance

**Fig. 4:**
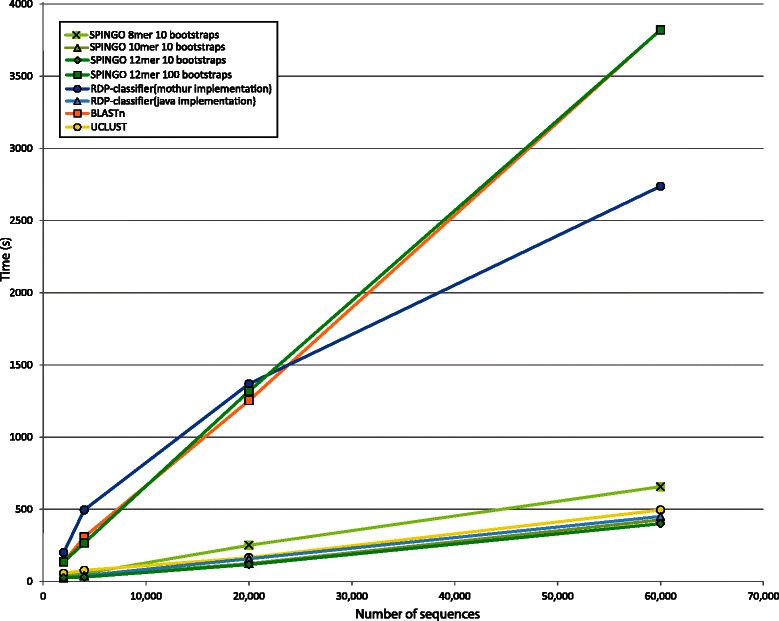
Comparison of time required to classify 16S rRNA gene V3V5 amplicon reads when trained on the SPINGO database. SPINGO run times using three different *k-mer* sizes and two different bootstrap values (8-mer with 10 bootraps by default) compared to the other methods all using *k-mer* 8. Only one CPU was used in all cases

SPINGO is a command-line, stand-alone and multi-threaded software package written in C++ with pre-and post-processing scripts written in Python. It can be run on a modest laptop requiring only 2 GB of RAM to accommodate the in-memory database for the default *k-mer* size. Processing time is inversely proportional to both the *k-mer* and database size, and is proportional to the sequence length. One million ~440 bp long V3V5 amplicons from the HMP sample analysed above were classified in ~1.7 h on a 64-bit server, utilizing 4 CPU threads and a *k-mer* size of 12. We compared SPINGO’s computational performance using three different *k-*mer sizes, with the other tested methods for species classification of HMP sample reads using their default settings. When only utilising one CPU for all methods we concluded that SPINGO using *k-*mer size 12was faster than all other methods (Fig. 4). While SPINGO with the default *8-*mer and 10 bootstrap settings is slower than UCLUST and Java-implementation of the RDP-classifier, it still outperforms the latter methods in terms of accuracy as outlined above. As expected, the 100 bootstrap setting does significantly slow SPINGO down, however only with a marginal improvement in accuracy (Additional file 2: Figure S1), thereby warranting the use of only 10 bootstraps when classifying large number of sequences.

## Conclusion

Here we present and demonstrate the utility and performance of SPINGO, a rapid, accurate and flexible classifier that improves the taxonomic resolution of 16S rRNA gene amplicons down to species level. While its primary target is species from any type of environmental sample, it can also be adapted to arbitrary classification hierarchies, like *Clostridium* clusters which are commonly used for characterising mammalian gut microbiota. SPINGO was consistently the most accurate species-classifier when compared to the other methods. To end with, the efficient algorithm provides a significant speed-up compared to existing classifiers which, when combined with its high accuracy, makes SPINGO a particularly valuable tool as amplicons more now than ever are sequenced in the hundreds of millions.

## Availability and requirements

The source code, executables and documentation are available at https://github.com/GuyAllard/SPINGO.

**Project name:** SPINGO

**Operating system(s):** Linux

**Programming language:** C++ / Python

**Other requirements:** To compile from source the following development libraries are required - Boost.program_options, Boost.serialization and Boost.thread

**License:** GNU GPL version 3

**Restrictions for use by non-academics:** None

## Additional files

Additional file 1:
**Additional information on how the 10-fold cross validation was performed, and how the reference databases were re-formated for use with compared methodologies.** (PDF 7 kb)
Additional file 2: Figure S1. The impact of *k-mer* size 8 and 12, as well as the impact of bootstrap values 10 and 100 on species level classification accuracy for SPINGO as shown by 10 fold cross validation. (PDF 312 kb)
