# Supplementary material for: SPINGO: a rapid species-classifier for microbial amplicon sequences
Source: BMC Bioinformatics. 2015 Oct 8;16:324. doi: 10.1186/s12859-015-0747-1 (PMC4599320; doi:10.1186/s12859-015-0747-1)
Supplement: Additional file 1: — Additional information on how the 10-fold cross validation was performed, and how the reference databases were re-formated for use with compared methodologies. (PDF 7 kb) [file 12859_2015_747_MOESM1_ESM.pdf]

10-fold cross validation.

This analysis was performed on the bash command line (bash version 4.2.37). It was done by randomly reorganising the SPINGO database using a combination of grep and shuf in bash. This new randomly reorganised database was split into tenths, where for each 10th the other 9/10ths were combined using cat. For each 10th, 12 different primer sets were used to extract the different variable regions to be used for classification using the v_ripper.py python script (https://github.com/GuyAllard/SPINGO). Following this, for each primer set, for each 10th of the database, the proportion of assignments which were correct was calculated and an average across all 10th of the database was determined as the classification accuracy for that primer set. This was then repeated for each of the other 3 classifiers, RDP-classifier (mothur v1.34.1), UCLUST (v1.2.22q) and BLASTn (v2.2.28).

Database reformatting

Of the four classification methods that were compared, SPINGO, RDP-classifier (mothur implementation) and UCLUST, require input of both a fasta file and a taxonomy file, the latter of which contains further information about the database. Each one also requires a unique layout for both the fasta headers and taxonomy file. BLASTn, although not specifically requiring a taxonomy file, will delimit both query and subject sequence name by whitespace characters, thus any spaces or tab characters in sequence names were replaced with dashes to allow the assignments to be easily compared to the original sequence header. Below is an example of bash code that can be used to reformat the SPINGO database to it compatible with any of the classifiers in this analysis.

```
sed -e 's/\t/-/g' -e 's/ /-/g' RDP_11.2.species.fa > RDP_11.2.species.blast.fa
```

<u>Reformatting for use with UCLUST</u>

```
cut -f 1 RDP_11.2.species.fa > RDP_11.2.species.gg.fa
```

```
cut -f 1,2 taxonomy.map | sed 's/\t/\tg__/' -e 's/\([a-z]\)_\([a-z]\)/\1;s__\2/' -e 's/$/;/' > taxonomy.map.gg
```

<u>Reformating for use with mother</u>

```
cut -f 1,2 taxonomy.map | sed -e 's/_/;/'  -e 's/$/;/'  > taxonomy.map.mothur
```

```
cut -f 1,2 RDP_11.2.species.fa > RDP_11.2.species.gg.fa
```

Creation of a cpn60db for use with SPINGO.

The cpn60 database was downloaded from http://www.cpndb.ca/cpnDB/home.php on March 4th 2015. Not all sequences within the database have a full species assignment, some a labelled as a genus, and others as unknown species. These were removed, providing 6690 full length cpn60 sequences which formed the cpn60 database for use in SPINGO. A taxonomy file was created from the headers of this file. This database and taxonomy was then reformatted as described above for use with the mothur implementation of the RDP-classifier.
