# Supplementary figures and images for: SPINGO: a rapid species-classifier for microbial amplicon sequences
Source: BMC Bioinformatics. 2015 Oct 8;16:324. doi: 10.1186/s12859-015-0747-1 (PMC4599320; doi:10.1186/s12859-015-0747-1)

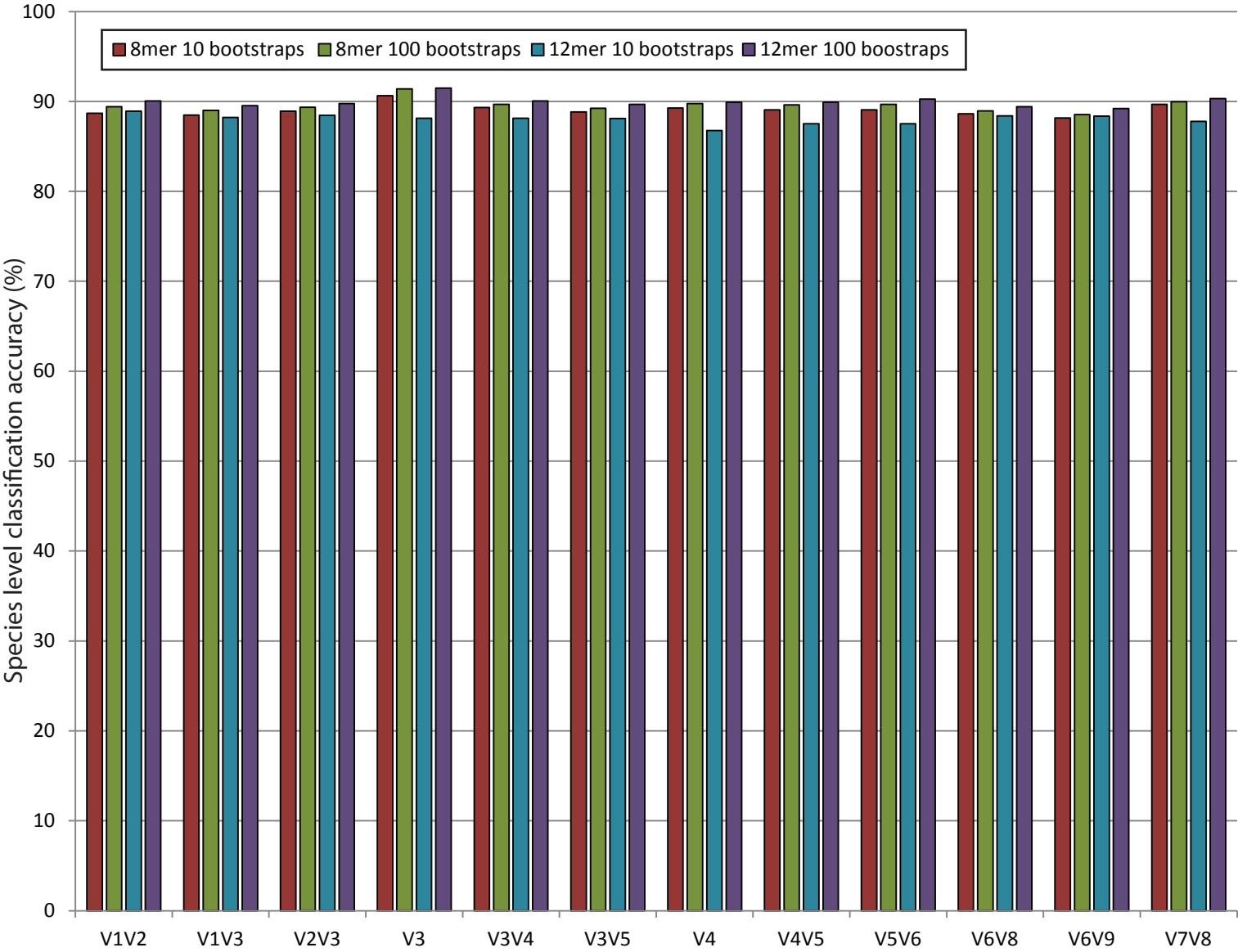

Supplement: Additional file 2: Figure S1. — The impact of k-mer size 8 and 12, as well as the impact of bootstrap values 10 and 100 on species level classification accuracy for SPINGO as shown by 10 fold cross validation. (PDF 312 kb) [file 12859_2015_747_MOESM2_ESM.pdf]
